# Cutaneous Adverse Reactions to COVID-19 Vaccines: Insights from an Immuno-Dermatological Perspective

**DOI:** 10.3390/vaccines9090944

**Published:** 2021-08-25

**Authors:** Dennis Niebel, Natalija Novak, Jasmin Wilhelmi, Jana Ziob, Dagmar Wilsmann-Theis, Thomas Bieber, Joerg Wenzel, Christine Braegelmann

**Affiliations:** Department of Dermatology and Allergy, University Hospital Bonn, 53127 Bonn, Germany; Natalija.Novak@ukbonn.de (N.N.); Jasmin.Wilhelmi@ukbonn.de (J.W.); Jana.Ziob@ukbonn.de (J.Z.); Dagmar.Wilsmann-Theis@ukbonn.de (D.W.-T.); Thomas.Bieber@ukbonn.de (T.B.); Joerg.Wenzel@ukbonn.de (J.W.); Christine.Braegelmann@ukbonn.de (C.B.)

**Keywords:** vaccines, COVID-19, adverse event, exanthema

## Abstract

(1) Background: Numerous vaccines are under preclinical and clinical development for prevention of severe course and lethal outcome of coronavirus disease 2019 (COVID-19). In light of high efficacy rates and satisfactory safety profiles, some agents have already reached approval and are now distributed worldwide, with varying availability. Real-world data on cutaneous adverse drug reactions (ADRs) remain limited. (2) Methods: We performed a literature research concerning cutaneous ADRs to different COVID-19 vaccines, and incorporated our own experiences. (3) Results: Injection site reactions are the most frequent side effects arising from all vaccine types. Moreover, delayed cutaneous ADRs may occur after several days, either as a primary manifestation or as a flare of a pre-existing inflammatory dermatosis. Cutaneous ADRs may be divided according to their cytokine profile, based on the preponderance of specific T-cell subsets (i.e., Th1, Th2, Th17/22, Tregs). Specific cutaneous ADRs mimic immunogenic reactions to the natural infection with SARS-CoV-2, which is associated with an abundance of type I interferons. (4) Conclusions: Further studies are required in order to determine the best suitable vaccine type for individual groups of patients, including patients suffering from chronic inflammatory dermatoses.

## 1. Introduction

**Figure 1:**
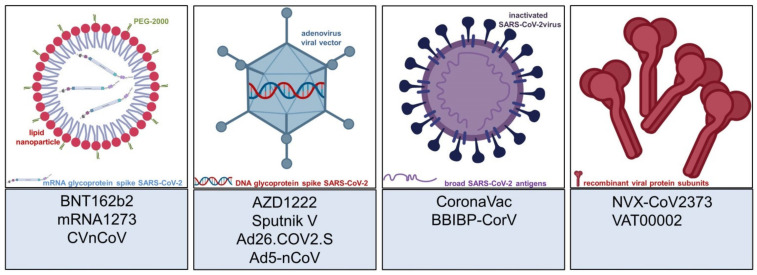
Most commonly used vaccine platforms and selected COVID-19 vaccines. From left to right: mRNA-based vaccines contain genetic information of the spike protein of SARS-CoV-2 in a lipid-nanoparticle-enveloped structure; viral vector vaccines contain DNA of the SARS-CoV-2 spike glycoprotein in a virus other than a coronavirus, most commonly an adenovirus; inactivated coronavirus vaccines contain a multitude of SARS-CoV-2 antigens, and are not limited to the spike glycoprotein; recombinant protein vaccines are specifically engineered molecules to evoke antiviral immunogenicity. Abbreviations: PEG—polyethylene glycol; mRNA—messenger ribonucleic acid; DNA—deoxyribonucleic acid; SARS-CoV-2—severe acute respiratory syndrome coronavirus 2.

The coronavirus disease 2019 (COVID-19) pandemic has led to the rapid invention and approval of vaccines against the responsible pathogen—severe acute respiratory syndrome coronavirus 2 (SARS-CoV-2) [1]. More than 100 companies and institutions worldwide developed vaccine candidates using both well-established and more experimental vaccine platforms [2]. Now, more than a year later, there are a variety of effective and safe COVID-19 vaccines, which are currently delivered worldwide. The already approved vaccines rely on nucleic-acid-based vaccine platforms—i.e., messenger ribonucleic acid (mRNA)—viral vector platforms (using different adenovirus strains), and inactivated virus. Protein subunit vaccines have not yet entered mass vaccination programs, but might follow in the near future (Table 1, Figure 1). Given the paucity of the vaccines and the urgent need for mass vaccination using billions of doses in light of the ongoing pandemic, there is significant geographical variety in the use of the different agents. Many countries lack access to COVID-19 vaccines, while some high-income countries already vaccinated the majority of their populations. We will consistently refer to the vaccines by their generic names, as the trade names may vary geographically.

A potent induction of antiviral immunity is achieved via humoral and cellular immune responses. To elicit sufficient immunogenicity, apart from those that use live attenuated virus, most vaccine types require repeated delivery and/or adjuvants to adequately spark the innate immune system to then elicit adaptive immune responses. Nucleic acids (including mRNA) represent danger-associated molecular patterns (DAMPs) that activate pattern recognition receptors (PRRs), including Toll-like receptors (TLRs), which mediate immunogenic effects [14]. Hence, the available COVID-19 mRNA vaccines do not require adjuvants [15]. The most commonly used adenovirus platforms are subject to a varying prevalence of pre-existing anti-vector immunity in the population if human strains are used [2]. However, they proved to be effective in achieving a durable response, and do not necessitate additional adjuvants; some of them are even intended for a single-dose regimen (e.g., Ad26.COV2.S and Ad5-nCoV). Non-human adenovirus strains are not subject to pre-existing host immunity (e.g., AZD1222), as the vector virus normally only affects chimpanzees. However, in principle, adjuvants deserve attention regarding vaccine-derived skin toxicity, as these agents bear the capacity to drive off-target inflammatory reactions [16]. After all, the skin is commonly involved in vaccine-derived adverse reactions [17]. This is an expected finding, since viral infections themselves may obligatorily produce characteristic cutaneous eruptions (e.g., measles—morbillivirus) or potentially produce paraviral cutaneous reactions (erythema multiforme—herpes simplex virus; Gianotti–Crosti syndrome—hepatitis B virus and others; papular pruritic gloves and socks syndrome—parvovirus B19 and others) [18]. Notably, infections with SARS-CoV-2 may also evoke particular cutaneous lesions in a minority of patients, such as vesicular, urticarial, maculopapular, and chilblain-like eruptions [19,20]. From a pathophysiological point of view, similar reactions might be seen upon immunogenic challenge with a corresponding vaccine. Conclusively, cutaneous adverse drug reactions (ADRs) seem to be frequent events in the course of COVID-19 vaccines, and include, among others, erythema, swelling, itching, pernio-like lesions, and generalized rashes (Table 1) [21]. Although these may be daunting for the patients and the treating physicians, in most clinical studies, they are not precisely reported from a dermatological point of view. For example, erythema, swelling, itch, or nodules are sometimes summarized as “local reaction”, while eczematous, vesicular, and morbilliform reactions are inconsistently summarized as “rash” (Table 1). Moreover, some more recent prospective “real-world” studies about ADRs to COVID-19 vaccines included reactions during the first week after vaccination or even only the first three days [22]. Only recently did dermatologists begin to report cutaneous ADRs with adequate distinction to reveal that the vaccines are capable of eliciting very different inflammatory cutaneous reactions [21,23,24]. It is also important to mention that the latency between vaccination and onset of delayed skin reactions may exceed 10 days [25]. Moreover, clinical trials normally exclude patients with pre-existing conditions requiring immunocompromising medication; hence, flares of pre-existing chronic inflammatory dermatoses might be underreported thus far [26].

**Figure 2:**
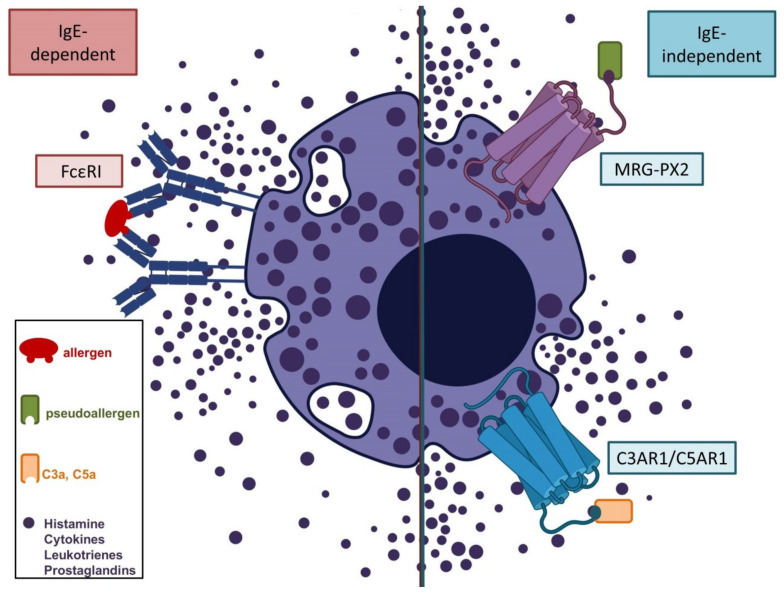
Supposed mechanisms of IgE-dependent (type I allergic) and non-IgE-dependent (pseudoallergic) immediate reactions to COVID-19 vaccines adapted from [27,30,32]. Type I allergic reactions occur due to dimerization of high-affinity IgE receptors (FcεRI) in sensitized individuals after contact with an allergen (e.g., PEGs). Non-IgE-dependent immediate reactions may occur via direct interaction of pseudoallergens with G-protein-coupled receptors (e.g., MRG-PX2), or as a result of complement activation (C3a, C5a) in individuals with specific IgG against components of the vaccine (e.g., anti-PEG IgG). Synchronized mast cell degranulation is the result of all three pathways, and causes an abrupt increase in blood levels of histamine, leukotrienes, prostaglandins, and other cytokines. Clinical symptoms such as angioedema, bronchial obstruction, and decreased blood pressure (shock) occur according to the extent of the anaphylactic/anaphylactoid reaction, and may be life-threatening.

We will herein summarize the current available reports of cutaneous ADRs in the course of different COVID-19 vaccines, and speculate about their pathophysiological backgrounds in light of the available data. We aim to highlight the variety of potential cutaneous inflammatory reactions in the course of vaccines to sharpen the focus of physicians who encounter such patients. Type I allergic reactions including anaphylaxis are beyond the scope of this article, and have been extensively reviewed previously [27,28,29,30]. Immunogenic and non-immunogenic immediate reactions and their pathophysiological mechanisms are briefly summarized in Figure 2. Comprehensive guidelines concerning safe vaccination of individuals with an allergic background have been established [31].

## 2. Literature Review

We searched for prospective and retrospective clinical studies, case series, and case reports about cutaneous ADRs to the most frequently deployed COVID-19 vaccines in PubMed (National Library of Medicine), up to 30 June 2021, to be included in the report. The search terms used were ‘vaccines’, ‘COVID-19’, ‘adverse event’, ‘exanthema’, ‘COVID-arm’, and further specific dermatological diagnoses, as mentioned in the discussion. Moreover, we screened the available data from the phase II/III trials of the respective vaccines.

## 3. Discussion

First of all, it is important to point out that serious cutaneous ADRs are very rare, and that the established vaccines have a satisfactory safety profile. Most of the encountered skin reactions are self-limiting, and require little or no therapeutic intervention. Local reactions include pain and erythema, and may be seen in a large proportion of the recipients; accordingly, they must be expected by patients and physicians. However, there are no exact numbers on the incidence of more unusual skin reactions. This is not surprising, as even the accepted incidence numbers of dermatoses in general are quite vague and depend largely on historic numbers. A recently published cross-sectional study from Spain precisely characterized the most commonly seen reactions from a dermatological point of view; however, the study design did not enable conclusions regarding the frequency in the respective population [33]. We compiled the available reports in another publication to estimate the incidence of generalized or more severe cutaneous ADRs to be below 0.3% [34]. Caution is necessary, as the underlying studies were subject to potential under- or over-reporting. In the light of mass vaccinations of general populations, low incidences still demand awareness by the treating physicians, who might encounter otherwise rare conditions more frequently. In the following paragraph we will clarify the general background of cutaneous ADRs, in order to then reflect on the available data on specific dermatoses.

**Figure 3:**
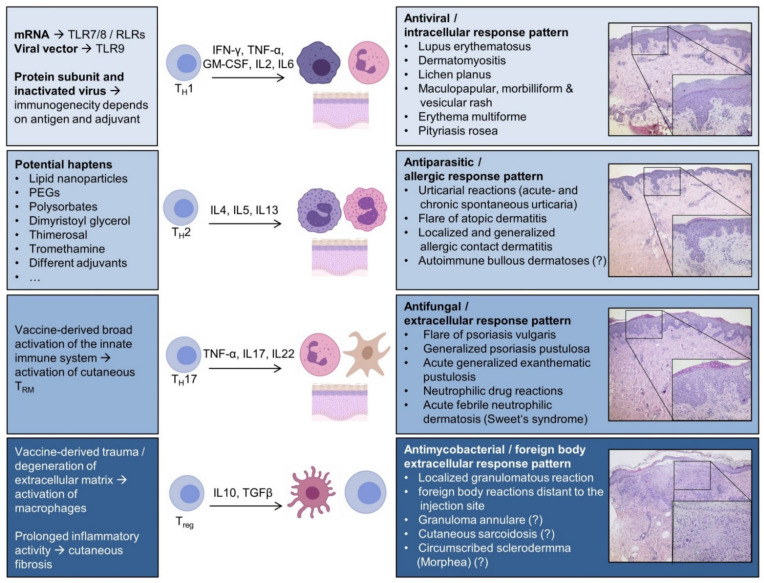
The mode of action varies among the different vaccine types, but it ultimately leads to increased expression of IFN-γ, which is a prerequisite for sufficient antiviral immunogenicity. Notably, mRNA and viral vector vaccines activate different TLRs; therefore, immunological differences appear plausible [36,37]. Moreover, vaccines comprise various molecules that potentially act as haptens to elicit type IV allergic reactions [38]. At this point, it is not clear whether specific vaccine types impose a larger risk for severe cutaneous ADRs to specific groups of patients, e.g., psoriatic patients. A dysregulation of regulatory T cells may shift macrophages to initiate granulomatous reactions, and longstanding inflammatory activity might induce fibrogenic alterations of the dermis to result in circumscribed scleroderma (morphea). Abbreviations: TLR—Toll-like receptor; RLR—RIG-I-like receptors; T_RM_—Tissue-resident memory T cell; PEG—polyethylene glycol. This immunological scheme is adapted from [35].

Immunogenic effects of vaccines lead to altered levels of chemokines and cytokines, which activate different key players of the innate and adaptive immune system (i.e., different T-cell and B-cell subsets, histiocytes/macrophages, dendritic cells, eosinophils etc.). The skin and mucosa as boundary surfaces to the environment are largely affected by the general activation of the immune system sparked by vaccines. According to the predominant type of cutaneous inflammation, one can differentiate at least four different patterns of inflammatory skin reactions [35] (Figure 3); that is, firstly a mainly cellular immune response pattern incorporated by CD8+ T cells and macrophages with a Th1-polarized T-helper cell profile. This reaction type is considered to be a classical antiviral/antitumor response, and would be a conclusive reaction to a trigger such as a vaccine. Key cytokines of this first reaction type are interferon-γ (IFN-γ), tumor necrosis factor-α (TNF-α), and different interleukins, including IL-2 and IL-6. Secondly, numerous components of vaccines may act as haptens to lead to a predominantly Th2-polarized inflammatory reaction with abundance of the hallmark interleukins IL-4 and IL-13. Additionally, cutaneous eosinophilia may be a feature in the course of IL-5 expression. Different atopic manifestations, including atopic dermatitis, are paradigmatic for this second inflammatory reaction. In case of prior sensitization to components of a vaccine, either an immediate anaphylactic reaction (type I allergic reaction) or a delayed allergic reaction (type IV allergic reaction) may occur, conclusively [27]. In the latter described situation, contact dermatitis may result as localized or generalized (hematogenous) eczema. Adjuvants might also play a key role in this inflammatory pattern, as aluminum may elicit a Th2 shift [2].

Thirdly, skin-resident memory T cells may be activated as a result of innate immune system activation in susceptible individuals, which ultimately elicits a Th17/Th22-predominant milieu. Preponderance of Th17/Th22 is typical for an inflammatory reaction to extracellular pathogens (e.g., dermatophytes, extracellular bacteria). An influx of neutrophils may then trigger both psoriasiform and pustular reactions. Fourthly, vaccine components may trigger inflammatory reactions that normally occur as a result of infections with mycobacteria, or as a response to foreign bodies, resulting in granulomatous reactions. This cascade is orchestrated by IL-10 and transforming growth factor beta (TGF-β) in the course of imbalance of proinflammatory and anti-inflammatory signals, causing macrophages/histiocytes to form granulomas. Additionally, vaccine-derived trauma and degradation of the extracellular matrix may initiate a fibrogenic inflammatory response, promoting connective tissue diseases such as circumscribed scleroderma (morphea) [35]. Other effects of vaccines may include immune complex formation, which is the etiopathological trigger of leukocytoclastic vasculitis of cutaneous capillaries and venules [39]. The spectrum of cutaneous vasculitides in the course of COVID-19 vaccines has been reviewed recently by another group, and will not be further discussed herein [40]. In the following sections, we will recapitulate the current point of view about the occurrence of distinct dermatoses in connection with COVID-19 vaccines.

### 3.1. Th1-Polarized Cutaneous Inflammation

#### 3.1.1. Cutaneous Lupus Erythematosus (CLE)

CLE can be triggered by endogenous or exogenous factors (including drugs and vaccines), and is considered to be a paradigmatic disease defined by Th1 polarization, with predominance of lesional type I interferons including IFN-γ [41]. However, when compared to systemic lupus erythematosus (SLE), there are only a few reports about new onset of CLE or lupus-like inflammation subsequent to vaccines, including measles [42] and influenza vaccines [43]. Flares of preexisting disease have been described in neonatal lupus upon the diphtheria, pertussis, and tetanus (DPT) combination vaccine [44]. We recently reported worsening disease in a patient with longstanding subacute CLE after the first dose of BNT162b2 [26]. Moreover, there are reports about Rowell’s syndrome (RS)—i.e., a rare subtype of CLE with features of erythema multiforme (EM)—or annular plaques resembling RS in the course of mRNA vaccines [34,45]. Notably, development of annular lesions has also been described with a COVID-19 vector vaccine, which could be interpreted as a lupus-like reaction in our opinion [46]. Perniones and chilblains are among the major cutaneous manifestations in individuals with mild COVID-19 infection, which might be attributable to a strong type I interferon response in young and otherwise healthy patients. Interestingly, similar skin lesions may also occur as a rare subtype of chronic CLE named “chilblain lupus erythematosus” (ChLE). Vascular dysregulation that derives from both cytokine overactivation and environmental factors is supposedly responsible for ChLE [41]. Recently, rapid onset of chilblains has also been described in the course of mRNA vaccines [47]; this vaccine-derived phenomenon is currently believed to resemble a mimic of the natural immune response to SARS-CoV-2 that also shows similarity to ChLE [21,48]. When compared to mRNA vaccines and vector vaccines, inactivated viral vaccines represent poor inducers of cytotoxic CD8+ T cells [2]. Speculatively, a decreased induction of Th1-polarized autoimmunity might be the result. However, the individual effects of inactivated virus vaccines largely depend on the respective virus and adjuvants.

Apart from the role of IFN-γ, there might be other CLE-triggering components in different COVID-19 vaccines. Gambichler et al. speculated about a potential role of PEGs, because similar lupus-like reactions have been described with PEGylated liposomal doxorubicin in the past [45]. Even though safety data are limited, vaccination against COVID-19 is desirable in almost all patients suffering from rheumatic conditions, including CLE patients [49,50]. Certain medications, including B-cell depleting agents (e.g., rituximab) or glucocorticosteroids in high doses, may interfere with vaccine efficacy, which must be considered. Helpful guidelines have been established to enable shared decision making with the affected patients [51].

#### 3.1.2. Dermatomyositis (DM)

DM and polymyositis represent a spectrum of autoimmune conditions defined by a self-directed attack towards structural proteins of skeletal muscle (autoimmune myopathy). DM may typically feature specific skin findings (heliotrope erythema, Gottron’s papules, mechanic’s hands etc.), and may be associated with internal malignancy. From a dermatological point of view, there are numerous similarities to subacute CLE, especially considering histopathology. Moreover, a drug-induced subtype of DM has been described, of which IFN-α is one of the potential triggers [52]. Until July 2021, there were no described cases of DM in the course of COVID-19 vaccines. Still, there is one report of vaccine-derived myositis at the injection site after receiving a COVID-19 vaccine [53]. As vaccines are delivered intramuscularly, associated trauma might predispose the individual to the development of an anti-muscular autoimmune or toxic reaction. Although not yet reported, such instances might be encountered.

#### 3.1.3. Lichen Planus (LP)

LP is a very common, mostly self-limiting dermatosis defined by an influx of CD8+ T cells to the skin and mucosa, resulting in pruritic papules and characteristic Wickham striae. Histologically, a “lichenoid” band of lymphocytes promotes inflammation along the dermo–epidermal junction, resulting in keratinocytic apoptosis (interface dermatitis). Although the etiopathology remains largely unknown, for years, viral infections including hepatitis B have been linked to this condition. Other triggers include drugs and vaccines (hepatitis B, influenza, rabies, and combination vaccines). Naturally, COVID-19 vaccines also bear the potential to elicit this particular skin disease, as recently described by Hiltun et al. [54]; the authors point out that the vaccine leads to increased levels of IL-2, TNF-α, and IFN-γ—the exact cytokines centrally involved in the development and perpetuation of LP. Therefore, more cases of de novo eruption of LP are to be expected with COVID-19 mass vaccinations.

#### 3.1.4. Maculopapular, Morbilliform, and Vesicular Rash

The term “rash” is commonly used in an unsatisfactory fashion. However, polymorphic exanthemas in connection with viral infections, drugs, or both may be specified as rash if a further description is added (e.g., morbilliform—“like an exanthema seen with measles”). Normally, paraviral exanthemas and maculopapular drug eruptions develop within one week, and are self-limiting; the same seems to be true for rashes associated with COVID-19 vaccines, assuming a correct diagnosis in the published data [21,55]. On the other hand, there are reports about very early onset of rashes—within hours—which then persist as pruritic eruptions over weeks [56]. Special attention is needed with purpuric rashes, as they may be an early sign of immune thrombocytopenic purpura. Thrombocytopenia as a cause of purpuric rashes may also be associated with immunogenic vascular hypercoagulability—the underlying factor of events such as sinus vein thrombosis. These are rare dangerous side effects that have been associated with adenoviral vector vaccines. It should be noted that there is also a report about mRNA-vaccine associated purpuric rash [57]. In our opinion, most maculopapular rashes associated with COVID-19 vaccines may be seen as an unspecific mimicry of skin findings associated with COVID-19, yet caution is advisable.

#### 3.1.5. Erythema Multiforme (EM)

EM is a characteristic skin eruption resembling target-like annular erythematous lesions, most commonly seen in children and young adults suffering from recurrent herpes simplex infections. Vaccines may trigger this characteristic dermatosis, which is most likely an immunogenic epiphenomenon to viral antigens [58]. Notably, EM has also been linked to the first dose of mRNA-1273 COVID-19 vaccine [21]. Finally, it should be noted that major-type EM is considered to be a continuous spectrum with life-threatening toxic anti-epithelial reactions (e.g., Stevens–Johnson syndrome, toxic epidermal necrolysis). The medical community should stay alert if such cases occur with COVID-19 vaccines, so as to define populations or ethnic groups at higher risk [40].

#### 3.1.6. Pityriasis Rosea (PR)

PR is a self-limiting pale exanthema along the Langer lines that originates from a preceding herald patch and commonly affects adolescents and young adults. It supposedly arises in the course of reactivation of human herpes virus 6 or 7 (HHV6/7); however, there are reports about development of PR both with COVID-19 infection and with COVID-19 vaccines, as published by Busto-Leis et al. [59]. Notably, both mRNA vaccines and viral vector vaccines seem to be potential causative agents. As with the etiology of PR itself, COVID-19-associated and COVID-19-vaccine-associated PR remain largely unexplained phenomena to date. Pre-existing immunity towards seasonal respiratory coronaviruses might designate “COVID-19-PR” as a paraviral epiphenomenon comparable to PR with HHV6/7 reactivation [59].

### 3.2. Th2-Polarized Cutaneous Inflammation

#### 3.2.1. Urticarial Reactions

Both acute urticaria and flares of pre-existent chronic spontaneous urticaria (CsU) seem to occur frequently during the first week after the first or second dose of COVID-19 vaccines [21]. This reaction might be the result of a raised susceptibility to mast cell degranulation in certain individuals. Potential reasons include an atopic background, occult chronic infections (e.g., helicobacter pylori), and drug insensitivities. Acute urticaria limited to the skin should not be confused with urticarial reactions with concomitant angioedema as a symptom of anaphylaxis, which typically affects multiple organs and may be life-threatening. Antihistamines may be advised liberally in these situations, as they are well tolerable and have few side effects. In the majority of patients, acute urticaria is a very straining but self-limiting condition that does not require further diagnostic workup. If symptoms do not wane within six weeks, CsU must be considered, and potential triggers should be identified.

#### 3.2.2. Atopic Dermatitis (AD)

As outlined above, flares of chronic inflammatory dermatoses, including AD, seem likely with COVID-19 vaccines, and have been reported occasionally [60,61]. This appears counterintuitive at first, but the chronically skewed cutaneous immune reaction in AD patients predisposes them to perpetuation of inflammatory loops sparked by different triggers. For example, shifting between Th1-polarized and Th2-polarized dermatoses with different treatments, including targeted biologicals, has been a topic of debate for some years now [62,63]. This phenomenon demonstrates the difficulty of rebalancing lost homeostasis in skin immunity. Regarding the most severely affected AD patients with established immunosuppressive treatments (e.g., dupilumab, baricitinib), there are now helpful position papers on optimal COVID-19 vaccine management [64].

#### 3.2.3. Injection Site Reactions and Allergic Contact Dermatitis

As outlined previously, pain and erythema are among the most frequent cutaneous ADRs to all COVID-19 vaccines. Furthermore, numerous components of vaccines may potentially act as haptens (among others: lipid nanoparticles in mRNA vaccines, PEGs, polysorbates, dimyristoyl glycerol, thimerosal, tromethamine) [27]. In previously sensitized individuals, reactivation of specific memory T cells quickly gives rise to an influx of various inflammatory cells, including Th2 cells. The result is an acute spongiotic dermatitis that exceeds the spectrum of a “normal” injection site reaction. As vaccine-derived allergens are injected into the deltoid muscle, hematogenous spread is much more likely to occur when compared to topical allergen exposure (e.g., occupational contact with latex); generalized eczematous reactions may be the consequence, accordingly. Some authors argue that delayed injection-site reactions (DIRs) and distant reactions involving cosmetic dermal fillers (e.g., hyaluronic acid) might be another manifestation of delayed hypersensitivity [65]. The phenomenon that has been dubbed “COVID-arm” has been linked initially to mRNA vaccines, especially mRNA-1273 [25,66,67]. On the other hand, more recent reports demonstrated comparable findings with viral vector vaccines as well [68]. Initial histological reappraisals of “COVID-arm” described a superficial and deep perivascular lymphocytic infiltrate with dilated vessels and intraluminal neutrophils [69]. Other authors found admixed eosinophils, which are typically involved in hypersensitivity reactions [70]. Further studies concerning the etiology of the underlying immune reaction of “COVID-arm” are warranted, and should be implemented into upcoming clinical trials involving mRNA vaccines. Moreover, it should be mentioned that “COVID-arm” may appear atypical in ethnic groups other than Caucasians [71]. Of note, deliberately using contact allergens in vaccines to boost immunogenicity is a concept that was proposed by a group from the UK in 2020 [72]. This concept might further enhance hypersensitivity reactions to vaccines and, accordingly, has not entered standard care.

#### 3.2.4. Autoimmune Bullous Dermatoses

Autoimmune blistering skin diseases are among the most severe dermatoses, and are potentially life-threatening. There is now an initial report about the initiation of pemphigus vulgaris in relation to an mRNA vaccine in an Asian woman [73]; the authors identified seven more cases of pemphigus vulgaris or foliaceus in connection with different vaccines (e.g., influenza). Therefore, the medical community ought to be watchful in this regard.

### 3.3. Th17-Polarized Cutaneous Inflammation

#### 3.3.1. Psoriasis Vulgaris

Similarly to AD, there are very limited data on COVID-19 vaccination in patients suffering from psoriasis vulgaris. Case reports exist about significant flares in close association with mRNA vaccines [74]. It is unclear at this point whether certain systemic treatments (conventional and biological disease-modifying antirheumatic drugs) affect the efficacy of COVID-19 vaccines, or whether these drugs diverge in the risk of a vaccine-derived boost of the underlying Th17/Th22 immune response, resulting in flare-ups. However, extensive reviews regarding this topic are available, and deduce a satisfactory safety profile with various biologicals [75]. For example, anti-IL-17 drugs are not expected to significantly impair the efficacy of COVID-19 vaccines based on experiences with influenza vaccines [76]. Interestingly, apremilast (a phosphodiesterase-4 inhibitor) has been associated with a low risk of flares of psoriasis, while at the same time enabling a sufficient vaccine immune response [77]. In spite of many uncertainties, there are helpful guidelines available to direct the clinician through vaccine management with his psoriatic patients. Vaccination is advisable for practically all psoriatic patients—especially those suffering from typical comorbidities, such as metabolic syndrome [78].

#### 3.3.2. Neutrophilic and Pustular Drug Reactions

Among the very severe cutaneous ADRs, there is a condition defined by rapid onset of innumerable pustules, called acute generalized exanthematous pustulosis (AGEP), which may be seen as a mimicker of generalized psoriasis pustulosa. Interestingly, both a case of AGEP associated with a viral vector vaccine [79] and a pustular flare of psoriasis associated with an inactivated virus COVID-19 vaccine [80] have been published recently. Another case report was classified as an overlap between AGEP and drug reaction with eosinophilia and systemic symptoms (DRESS) associated with a viral vector vaccine [81]. Another dermatosis may arise with succulent erythematous plaques and fever, which is called acute neutrophilic dermatosis (Sweet’s syndrome), and is also described in the course of both influenza vaccination [82] and COVID-19 mRNA vaccination [83].

As neutrophilic dermatoses might be largely unknown to health care professionals other than dermatologists, we believe that severe cutaneous ADR should be handled by specialized institutions.

### 3.4. Granulomatous and Fibrogenic Reactions

Although adverse reactions distant from the injection site are commonly described as hypersensitivity reactions, granulomatous tissue reactions might also be an etiopathological factor, especially with cosmetic dermal fillers [84]. Generation of granulomas is a physiological tool to limit the extension of pathogens or foreign bodies that cannot be eliminated. Another interesting phenomenon was described by Lopatynsky-Reyes et al. in terms of local skin inflammation of BCG vaccine scars after COVID-19 vaccines in health care workers [85]. Although there are no published case reports at this time, other specific granulomatous dermatoses such as granuloma annulare or cutaneous sarcoidosis might occur with COVID-19 vaccines, as they have been described in the course of other vaccines [86,87]. The same applies to circumscribed scleroderma (morphea), which was described in relation to vaccines in the past [88]. Similar to other inflammatory dermatoses, the potential risk of worsening of preexisting granulomatous or fibrogenic conditions should not deter the delivery of vaccinations to at-risk patients [89].

### 3.5. Further Implications, Unanswered Questions, and Future Challenges

In the available reports, there is a striking female predisposition for cutaneous ADRs. Possible reasons for this finding include reporting bias, as well as the fact that predominantly female health care workers were the first to receive vaccines in most countries [21]. On the other hand, women have a higher incidence of autoimmune diseases, and might be genuinely at higher risk for vaccine-derived ADRs [21]. Moreover, there are sex differences in vaccine-induced immunity that might be partly responsible for inequality of outcomes between women and men [90]. This is just one of many questions that need to be addressed in future studies. More clarification about the exact incidence of specific ADRs, including cutaneous ADRs, is desirable for distinct groups of patients (e.g., juveniles and elderly population) with each vaccine. How different types of COVID-19 vaccines differ in their safety profiles, and whether certain ethnic groups are at higher risk for specific side effects, should also be investigated. At this point, it is largely inconclusive as to how to proceed with the vaccination if serious non-allergic skin reactions occurred after the first dose, not to mention the optimal management of such cutaneous ADRs. In view of potential further mutations of SARS-CoV-2 that might require third/fourth or even yearly booster doses, excellent tolerability of vaccines is paramount for successful vaccination campaigns. The risk of sensitization to singular or multiple components must also be incorporated. Another puzzling detail is the stark variability in the time of onset of actual similar skin findings (e.g., maculopapular rashes). Clearly, dermatologists should be involved in future clinical trials in order to gain more immuno-dermatological insights into cutaneous ADRs to vaccines. A detailed and standardized reporting of “real-world” events would enable a better understanding of underlying pathophysiological mechanisms, and should therefore be implemented. Moreover, it will be important to define the most effective and tolerable vaccines for subsets of patients; this might include the combined use of different vaccine types. Furthermore, specific alterations of “first-generation” COVID-19 vaccines might help to enable better tolerance for patients at risk of ADRs.

Based on our lack of experience, we cannot draw conclusions regarding cutaneous side effects of vaccines that have been developed in Russia and China, as they are not yet approved for use in Germany. Singular case reports and case series about cutaneous side effects of these agents are available [80,91,92]. A group from the USA has published a comprehensive overview of cutaneous ADRs to various vaccines that deserves mention [93].

## 4. Conclusions

COVID-19 vaccines may evoke numerous delayed cutaneous ADRs, either de novo or in terms of a flare of pre-existing dermatosis. These reactions may be divided according to their cytokine profiles, based on the preponderance of specific T-cell subsets. Etiopathological triggers include an abundance of type I interferons as a direct effect of the immunogenicity of vaccines (Th1 response), delayed-type hypersensitivity reactions to components of the vaccines (Th2 response), activation of tissue-resident memory T cells in susceptible individuals (Th17/Th22-response), and vaccine-derived damage to the extracellular matrix (imbalance of regulatory T cells). Moreover, some cutaneous ADRs to vaccines (including vesicular/urticarial reactions or chilblains) may be seen as mimics to the natural infection with SARS-CoV-2, as the correspondent vaccine elicits similar immunogenic mechanisms. Naturally, the time of vaccine delivery and the onset of symptoms may be pure coincidence; however, the mere frequency of otherwise rare inflammatory skin diseases points towards a connection [94]. More generally speaking, vaccines may “awake the sleeping dragon” of specific autoimmune reactions, including inflammatory skin reactions, in vulnerable patients [95]. In light of the ongoing pandemic and the emerging features of SARS-CoV-2 variants, potential side effects of COVID-19 vaccines should not impose an obstacle to the delivery of these powerful tools in the fight against the virus. However, awareness of rare side effects is necessary for health care professionals. Dermatologists are centrally involved in the diagnosis and treatment of cutaneous ADRs, and should be prepared to counsel their patients accordingly [96].

## Figures and Tables

**Table 1:** Selection of COVID-19 vaccines and the most common ADRs, with a focus on cutaneous side effects, as of July 2021. Pain or tenderness at the injection site is very common with all available agents, and is therefore excluded in this tabular overview. Please note that further vaccines have entered clinical trials [3]. Abbreviations: mRNA—messenger ribonucleic acid; UK—United Kingdom; USA—United States of America; DRESS—drug reaction with eosinophilia and systemic symptoms.

Vaccine (Developing Institution)	Vaccine Type	First Approval	General ADRs	Cutaneous ADRs
BNT162b2 *Tozinameran, Comirnaty* (BioNTech/Pfizer)	mRNA	December 2020 in UK	>10%: Fatigue, headache, musculoskeletal pain, fever [4]	1–10%: Local injection site reaction: erythema, swelling; <1%: delayed local reactions (“COVID-arm”), morbilliform rash, urticarial reactions, pityriasis rosea; singular cases: Rowell’s syndrome, lichen planus
mRNA-1273 *Spikevax* (Moderna)	mRNA	December 2020 in USA	>10%: Fever, headache, fatigue, myalgia, arthralgia, nausea, chills [5]	1–10%: Local injection site reaction: erythema, swelling; <1%: delayed local reactions (“COVID-arm”), morbilliform rash, urticarial reactions, pityriasis rosea, erythema multiforme, erythromelalgia, herpes simplex, herpes zoster, perniones/chilblains; singular cases: reactions to cosmetic fillers, purpuric/petechial rash
AZD1222/ChAdOx1 nCoV-19 *Vaxzevria, Covishield* (AstraZeneca)	Viral vector vaccine (adenovirus)	December 2020 in UK	>10%: Fatigue, nausea, musculoskeletal pain, headache, subfebrile temperatures [6]	1–10%: Local injection site reaction: erythema, swelling; <1%: itch, rash, sweating; singular cases: psoriasis, rosacea, vitiligo, Raynaud’s phenomenon, cellulitis, pityriasis rosea, delayed large local reactions
Gam-COVID-Vac/Sputnik V (Gamaleya Research Institute)	Viral vector vaccine (adenovirus)	August 2020 in Russia	1–10%: Flu-like illness, headache, asthenia [7]	1–10%: Local injection site reactions, not further specified; <1%: indeterminate rash, petechial rash, “allergic rash”, itching, eczema/dermatitis; singular cases: abscess, alopecia, acneiform dermatitis
Ad26.COV2.S/JNJ-78436735 *COVID-19 Vaccine Janssen* (Johnson & Johnson)	Viral vector vaccine (adenovirus)	February 2021 in USA	>10%: fatigue, headache, myalgia, nausea, pyrexia [8]	1–10%: Local injection site reaction: erythema, swelling; singular cases: widespread annular eruption, DRESS-syndrome
Ad5-nCoV *Convidecia* (CanSinoBIO)	Viral vector vaccine (adenovirus)	February 2021 in China	>10%: fatigue, fever, headache, muscle pain, joint pain [9]	1–10%: Local injection site reaction: redness, swelling, itch; singular cases: non-infective gingivitis, buccal ulcerations, herpes simplex
CoronaVac (Sinovac)	Inactivated whole virus (aluminum adjuvant)	February 2021 in China	1–10%: fatigue, diarrhea, fever, muscle pain, headache, nausea, cough [10]	1–10%: Local injection site reaction: swelling, redness, pruritus, discoloration, induration; <1%: urticaria, petechial rash, flare of pustular psoriasis
BBIBP-CorV (Sinopharm)	Inactivated whole virus (aluminum adjuvant)	December 2020 in China	1–10%: fever, fatigue, inappetence, nausea, constipation, headache [11]	1–10%: Local injection site reaction: erythema, swelling, induration, “mucocutaneous abnormalities”; <1%: “rash”, itch, herpes simplex, buccal ulcer
NVX-CoV2373 (Novavax)	Recombinant protein subunit (saponin adjuvant)	Not yet approved	>10%: arthralgia, fatigue, headache, myalgia, nausea, malaise [12]	1–10%: Local injection site reaction: erythema, induration or swelling
CVnCoV *Zorecimeran* (CureVac)	mRNA	Not yet approved	“Dose dependent effects included fever, headache, fatigue, chills, myalgia, arthralgia, nausea/vomiting, diarrhea” [13]	1–10% local injection site reaction: swelling and itching (preliminary data)
VAT00002 *Sanofi–GSK COVID-19 vaccine* (Sanofi/ GlaxoSmithKline)	Recombinant protein subunit (AS03 adjuvant)	Not yet approved	No data available, yet (NCT04762680) Phase III trial launched in May 2021	No data available, yet

## Data Availability

Data sharing is not applicable to this article, as no datasets were generated or analyzed during the current study.
